# Dual-Prep registry: atherectomy devices and intravascUlAr lithotripsy for the PREParation of heavily calcified coronary lesions registry

**DOI:** 10.1007/s12928-025-01130-9

**Published:** 2025-05-12

**Authors:** Masato Nakamura, Nehiro Kuriyama, Yutaka Tanaka, Seiji Yamazaki, Tomohiro Kawasaki, Takashi Muramatsu, Kazushige Kadota, Takashi Ashikaga, Akihiko Takahashi, Satoru Otsuji, Kenji Ando, Masaru Ishida, Shigeru Nakamura, Yoshiaki Ito, Raisuke Iijima, Gaku Nakazawa, Junya Shite, Junko Honye, Junya Ako, Hiroyoshi Yokoi, Ken Kozuma, Hiromasa Otake, Kazuho Masumura, Tomomi Yamada, Yohei Sotomi

**Affiliations:** 1 Division of Minimally Invasive Treatment in Cardiovascular Medicine, Toho University Ohashi Medical Center, 2-22-36, Ohashi, Meguro-Ku, Tokyo, 153-8515 Japan; 2 Department of Cardiology, Miyazaki Medical Association Hospital, Miyazaki, Japan; 3 Department of Cardiology, Shonan Kamakura General Hospital, Kanagawa, Japan; 4 Department of Cardiology, Sapporo Higashi Tokushukai Hospital, Hokkaido, Japan; 5 Department of Cardiology, Tenjinkai Shin-Koga Hospital, Fukuoka, Japan; 6 Department of Cardiology, Fujita Health University Hospital, Aichi, Japan; 7 Department of Cardiology, Kurashiki Central Hospital, Okayama, Japan; 8 Department of Cardiology, Japanese Red Cross Musashino Hospital, Tokyo, Japan; 9 Department of Cardiology, Sakurakai Takahashi Hospital, Hyogo, Japan; 10 Department of Cardiology, Higashi Takarazuka Satoh Hospital, Hyogo, Japan; 11 Department of Cardiology, Kokura Memorial Hospital, Fukuoka, Japan; 12 Division of Cardiology, Department of Internal Medicine, Iwate Medical University, Iwate, Japan; 13 Department of Cardiology, Kyoto Katsura Hospital, Kyoto, Japan; 14 Department of Cardiology, Saiseikai Yokohamashi Tobu Hospital, Kanagawa, Japan; 15 Department of Cardiovascular Medicine, Toho University Ohashi Medical Center, Tokyo, Japan; 16 Department of Cardiology, Kindai University Hospital, Osaka, Japan; 17 Department of Cardiology, Osaka Saiseikai Nakatsu Hospital, Osaka, Japan; 18 Department of Cardiology, Kikuna Memorial Hospital, Kanagawa, Japan; 19 Department of Cardiovascular Medicine, Kitasato University Hospital, Kanagawa, Japan; 20 Cardiovascular Center, Fukuoka Sanno Hospital, Fukuoka, Japan; 21 Department of Cardiology, Teikyo University Hospital, Tokyo, Japan; 22 Graduate School of Medicine/Division of Cardiovascular Medicine, Kobe University, Hyogo, Japan; 23 Department of Medical Innovation, Osaka University Hospital, Osaka, Japan; 24 Department of Cardiovascular Medicine, Osaka University Graduate School of Medicine, Osaka, Japan

**Keywords:** Calcification, Atherectomy, Lithotripsy, Drug-eluting stent, OCT

## Abstract

Evaluation of calcified lesions by intravascular imaging has revealed that atherectomy devices have only limited impact. However, subsequent use of coronary intravascular lithotripsy (IVL) may increase treatment effectiveness without increasing risk of complications. This study was designed to evaluate the safety and effectiveness of IVL use after atherectomy in severely calcified coronary lesions as pre-treatment for drug-eluting stents (DES). The Dual-Prep registry is a multicenter, prospective registry of consecutive image-guided percutaneous coronary interventions (PCI). The primary effectiveness and safety endpoints were procedural success (residual stenosis < 50% by quantitative coronary angiography) without an in-hospital major adverse cardiac event (MACE) and 30-day freedom from MACE, respectively. Baseline vessel calcification score and final DES expansion were evaluated by optical coherence tomography (OCT). A total of 118 patients with 120 lesions were enrolled at 20 sites. The calcification score of lesions after atherectomy by core-lab assessment was 4.0 in all cases. Rotational atherectomy was applied prior to IVL in 83.9% cases with mean burr size of 1.57 ± 0.20 mm; IVL was subsequently successfully delivered in all cases (mean balloon diameter 3.02 ± 0.45 mm), followed by DES deployment (mean diameter 3.19 ± 0.51 mm, length of 36.3 ± 16.0 mm). The primary efficacy and safety endpoints were met in 98.3% and 98.3% of cases, respectively. A DES expansion index < 0.8 was seen in 42.2%, and an eccentricity index < 0.7 was not observed in any patient. In severely calcified lesions, image-guided atherectomy followed by IVL lesion preparation demonstrated high procedural success rates and satisfactory non-eccentric stent expansion. This approach may be considered for lesions where an ‘IVL-first’ strategy may not be feasible. jRCT1032230384 (Oct 7, 2023).

**Graphical abstract:**

Dual-Prep registry : atherectomy + IVL before DES implantation strategy for calcified lesion (Calc score ≥ 3 after atherectomy) 
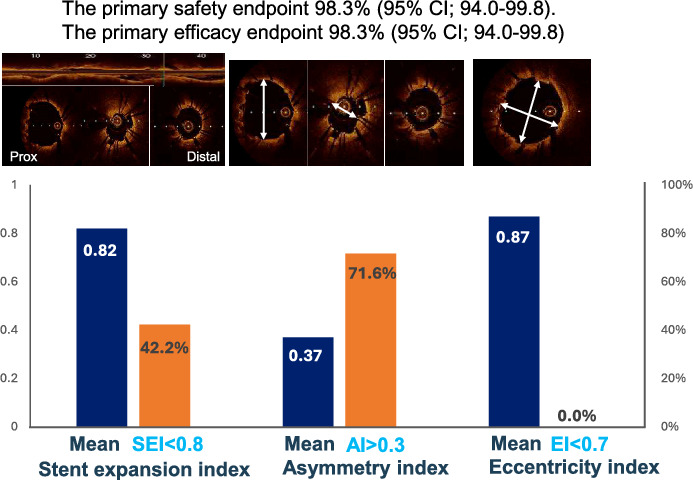

**Supplementary Information:**

The online version contains supplementary material available at 10.1007/s12928-025-01130-9.

## Introduction

The opportunity to treat severely calcified coronary artery lesions has increased with the aging of the population and the increased prevalence of diabetes mellitus and chronic kidney disease. This trend is expected to continue [1, 2]. Although outcomes of percutaneous coronary intervention (PCI) have improved remarkably with the advent of drug-eluting stents (DES), PCI for complex lesions, including those with severe calcification, remains challenging, even in the DES era. Initial success rates are lower and long-term clinical outcomes appear less favorable, leaving considerable room for improvement [3, 4]. Consensus documents recommend a stent expansion index of > 0.8, and inadequate stent expansion is a known determinant of stent failure by restenosis or thrombosis [5]. Accordingly, pre-treatment to modify calcified lesions is considered a key element in the DES treatment of calcified lesions [6, 7]. The efficacy and safety of rotational atherectomy (RA), orbital atherectomy (OA), intravascular lithotripsy (IVL), and modified balloon as lesion preparation devices have been validated [8]. Nevertheless, these devices have demonstrated limited impact on heavily calcified coronary lesions [8], and room for further investigation remains. In fact, a recent analysis of the outcomes of contemporary PCI in imaging guides found that calcified lesions remain an independent risk factor against revascularization [9].

The efficacy of atherectomy techniques may be determined by the presence of guidewire bias, which may limit the effectiveness of such tools. Additionally, while aggressive atherectomy has been proposed to improve stent expansion, findings to date have not demonstrated an advantage for this approach, but rather shown associations with increased angiographic complications and poorer clinical outcomes [10, 11]. IVL is a guidewire bias-independent device but may have disadvantages in crossability, particularly in chronic total occlusion and in more complex, tortuous, long and calcified lesions. The combination of atherectomy and IVL, therefore, appears a reasonable approach that might increase the efficiency of complex lesion treatment without compromising safety. Of note, the previous studies did not include imaging-guided treatment strategies, which has hindered accurate assessment of this approach. Intracoronary imaging guidance allows direct and detailed assessment of lesion preparation efficacy before stenting, and may provide deeper insight into the effectiveness of IVL following standard atherectomy. For these reasons, it has been granted an indication in recent European guidelines for the management of complex coronary disease [12].

In this study, we evaluated the efficacy and safety of combining atherectomy and IVL in image-guided DES implantation in heavily calcified complex coronary lesions.

## Methods

### Study design

DUAL-PREP is a prospective, multicenter, single-arm study designed to assess the safety and efficacy of combination use of atherectomy devices and IVL before DES deployment in patients with severely calcified lesions. Inclusion criteria were (1) age ≥ 18 years; (2) consent to participate in the study; (3) presence of severely calcified lesions for which treatment with IVL after other atherectomy devices may be preferable, based on imaging findings; and (4) confirmation of severe calcification, defined by angiography showing radiopaque images without cardiac motion prior to contrast injection involving both sides of the arterial wall in at least one location with ≥ 15 mm total calcification length and extending partially into the target lesion [13]. The severity of calcification was also confirmed by OCT/OFDI.

Exclusion criteria were (1) participation in other clinical trials that may affect the results, and (2) ineligibility for treatment with atherectomy or IVL. Designated atherectomy devices were the ROTAPRO™ Rotational Atherectomy System (Boston Scientific, Marlborough, Massachusetts) and Diamondback 360™ Coronary Orbital Atherectomy System (Abbott Vascular, Santa Clara, California). The Shockwave C^2^ Coronary IVL system (Shockwave Medical, Santa Clara, California) was utilized for IVL. The study protocol was approved by central review (Institutional Review Board of Toho University Ohashi Hospital Ethics Board H23024 H23 May 22, 2023). Study design details are summarized in supplemental Table 1.

### Study procedures

Imaging evaluation of coronary lesions with OCT/OFDI was scheduled at least four times, namely before lesion instrumentation, after RA/OA, after IVL, and after stent deployment.

The basic sequential treatment strategy was as follows.

- RA was conducted in cases with a preoperative imaging evaluation calcification score greater than 3 or failure of a cross imaging catheter.
- RA burr size was typically selected, such that the burr-to-artery ratio did not exceed 0.7. Rotational speed ranged 140,000 to 200,000 rotations per minute [14].
- For OA, a 1.25-mm classic crown burr was used. All patients were initially treated with a low speed (8000 rpm). Speed was increased to high (120,000 rpm) when the OCT pullback demonstrated that the guidewire was not attached to the normal vessel wall in the lesion, and tissue modification did not extend beyond the media. The choice between OA and RA was at the operator’s discretion.
- After atherectomy (RA/OA), IVL was set for use when a calcified lesion was considered inadequately pretreated, but further atherectomy was inappropriate, e.g., slow flow, deep calcification, or when guidewire bias limited atherectomy effectiveness.
- DES deployment after IVL dilation.
- Additional balloon dilatations before or after IVL and before or after DES deployment was at the operator's discretion.

The details of the recommended procedure are in supplement Fig. 1.

**Fig. 1:**
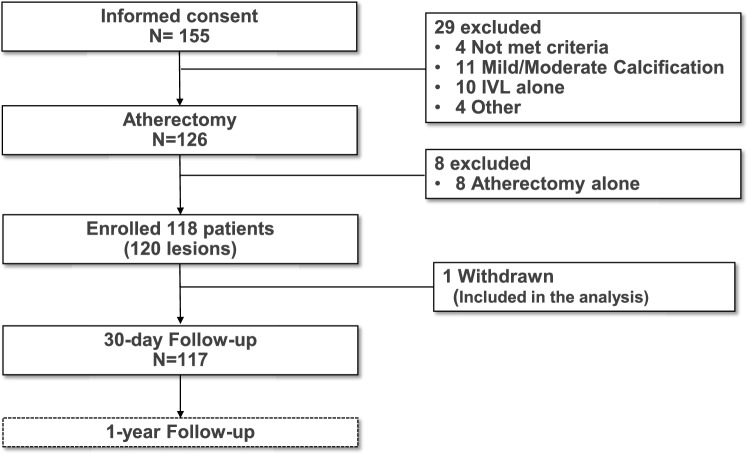
Study flowchart. Patients was enrolled from 20 sites in Japan through Nov 2023 to June 2024. *IVL* Intravascular Lithotripsy

### Study endpoints

The primary safety endpoint was freedom from major adverse cardiac events (MACE) within 30 days of the procedure, defined as a composite of cardiac death, myocardial infarction (MI), or target vessel revascularization (TVR). MI was classified into periprocedural MI and spontaneous MI, with the former adjudicated according to the Society for Cardiovascular Angiography and Interventions (SCAI) definition [15] and the latter by the 4th Universal definition [16]. The primary efficacy endpoint was procedural success, defined as residual stenosis < 50% after stenting (core laboratory assessment) without MACE during hospitalization. Angiographic success was defined as successful IVL crossing, balloon expansion & therapy delivery, residual stenosis ≤ 30% after stenting, and no serious angiographic complications. An independent Clinical Evaluation Committee (CEC) evaluated all clinical events. Prespecified endpoints and definitions are summarized in supplemental Table 2.

### Angiographic evaluation

Angiography and OCT/OFDI were analyzed in the core lab (Micron, Inc. Osaka, Japan). Quantitative coronary angiography was performed by coronary angiography before the procedure, after atherectomy, after IVL, and after stenting. All analyses were performed offline using QAngio XA 7.3.102.0 (Medis Medical Imaging System, Leiden, the Netherlands). Coronary angiography after vasodilator administration was performed in the projection with the highest degree of stenosis, and consecutive evaluations were performed in the same projection. Minimum lumen diameter, % diameter stenosis, and lesion length were calculated. Severe angiographic complications were defined as abrupt occlusion, coronary perforation, persistent slow flow/no reflow, and type D-F (flow-limiting) coronary artery dissection.

### OCT/OFDI evaluation

OCT/OFDI was recorded using the Dragonfly OpStar™ imaging catheter and OPTIS imaging system (Abbott Vascular, Santa Clara, California) or FastView® imaging catheter and LUNAWAVE® imaging system (Terumo Corporation, Tokyo, Japan). During image acquisition, 8–14 mL of nondiluted iodinated contrast or low-molecular-weight dextran was injected at a rate of 3–4 mL/s to achieve blood clearance, and an automated pullback system at a speed of 36 mm/s was used. OCT/OFDI images were analyzed offline using the AptiVue™ ORW (Abbott Vascular, Santa Clara, California)/LUNAWAVE® Offline Viewer Software ver.1.2 (Terumo Corporation, Tokyo, Japan) at 1-mm intervals in accordance with expert consensus reports [15]. Calcified plaques were evaluated as areas of heterogeneity, low internal intensity, low attenuation, and well-defined boundaries. Calcified plaques were systematically scored as previously described [5], with 2 points for maximum angle > 180°, 1 point for maximum thickness > 0.5 mm, and 1 point for length > 5 mm. Stent expansion index was defined as the ratio of the minimal stent area to the ideal lumen area, calculated using a linear model derived from the proximal and distal reference areas. Asymmetry index [17, 18] and eccentricity index [19] were calculated as previously described.

### Statistical analysis

Based on analysis of previously published, real-world atherectomy studies, a minimum of 110 evaluable patients would provide sufficient statistical power to assess the primary safety endpoint. This sample size would enable evaluation of a 90% MACE-free rate with a 95% confidence interval ranging from 82.4 to 94.7% [10, 20–22]. Continuous variables are expressed as mean ± SD. Categorical variables were summarized as frequencies and portions. Point estimates and Clopper–Pearson 95% confidence intervals (CIs) were calculated for the primary endpoint. Cumulative frequency curves of angiographic parameters were obtained to assess the effect of consecutive procedures. Analyses were performed by an independent biostatistician using SAS version 9.4 statistical analysis software (SAS Institute Inc., Cary, NC, USA).

## Results

Informed consent was obtained from 155 patients at 20 participating centers between November 2023 and June 2024. All patients were enrolled at the time of attempted treatment with the IVL system after use of atherectomy, regardless of whether the IVL catheter reached the lesion or not. A total of 118 patients with 120 lesions were enrolled, after excluding cases with no severe calcification on imaging and those treated with either IVL or atherectomy alone. A flowchart of the study is shown in Fig. 1.

Patient demographics and lesion characteristics are shown in Table 1. Mean age was 75.8 ± 8.9 years, 70.3% of patients were male, 56.8% had diabetes mellitus, and 25.4% were on hemodialysis. The majority of patients presented with chronic coronary syndrome, and 91.5% had de novo coronary artery lesions. All lesions were ACC/AHA type B2/C and exhibited severe angiographic calcification. Main target vessel was the left anterior descending artery, in 64.2% of cases. Mean reference vessel diameter was 2.67 ± 0.69 mm and mean lesion length was 34.3 ± 15.2 mm. Procedural factors are described in Table 2. A transradial approach was applied in 63.5% of cases. Preprocedural evaluation by intracoronary imaging was possible in 56.7% and gave a calcification score of 4.0 ± 0.0. RA was performed in 83.9% of patients; mean burr size was 1.57 ± 0.20 mm, and 8.5% had a larger burr size used as the second procedure. The remaining 16.9% underwent OA. Even after atherectomy, the calcification score of lesions on repeat intracoronary imaging remained at 4.0 in all cases. The main reasons for subsequent IVL use (multiple responses allowed) were concerns about the safety of additional atherectomy in 42.4% of cases, an expected lack of benefit with additional atherectomy in 60.2%, and other reasons in 1.7%. IVL was performed mainly with an IVL size of 2.5 or 3.0 mm. All patients had successful passage of IVL across the target lesion. A DES was implanted in all cases, with a mean diameter of 3.19 ± 0.51 mm and length of 36.3 ± 16.0 mm. DES was deployed directly following IVL in 57.5% of cases and following additional balloon post-dilatation after IVL in the remainder. Guide extension catheters were used in 47.5% of cases at the operator’s discretion. One long lesion was treated using a hybrid approach of proximal DES and distal DCB application. DES post-dilatation was performed in 79.2% of cases, with a mean maximum balloon diameter of 3.5 mm and a mean inflation pressure of 17.9 atmospheres.

**Table 1:** Patient demographics and lesion characteristics

Patient demographics	n = 118 (%)
Age (years)	75.8 ± 8.9
Male	83 (70.3)
Body weight (kg)	60.3 ± 12.9
Body Mass Index	23.3 ± 3.9
Diabetes mellitus	67 (56.8)
Hypertension	97 (82.2)
Hyperlipidemia	91 (77.1)
Current smoker	7 (5.9)
Clinical presentation	
Stable angina pectoris	80 (67.8)
Acute coronary syndrome	12 (10.2)
Silent ischemia	26 (22.0)
Previous myocardial infarction	21 (17.8)
Previous stroke	17 (14.4)
Previous PCI	44 (37.3)
History of coronary artery bypass graft	4 (3.4)
Atrial fibrillation	17 (14.4)
De novo lesion	108 (91.5)
Ejection fraction	57.0 ± 10.7
eGFR	45.1 ± 27.0
Hemodialysis	30 (25.4)

Lesion characteristics	n = 120 (%)
Lesion location	
Right coronary artery (RCA)	37 (30.8)
Left descending artery (LAD)	77 (64.2)
Left circumflex artery (LCX)	4 (3.3)
Left main trunk (LMT)*	6 (5.0)
Chronic total occlusion	2 (1.7)
Bifurcation lesion	31 (25.8)
Calcium nodule	50 (41.7%)
Severe calcification on angiography	120 (100)
Type B2/C	120 (100)
Reference vessel diameter (mm)	2.67 ± 0.69
Minimum lumen diameter (mm)	0.72 ± 0.28
Diameter stenosis (%)	72.6 ± 9.6
Lesion length (mm)	34.3 ± 15.2

*eGFR* estimated glomerular filtration rate, using the MDRD formula

*LMT includes three lesions for LMT-LAD and one lesion for LMT-LCX

**Table 2:** Procedural characteristics

Procedural characteristics	
Radial approach	75 (63.6)
Transfemoral approach	38 (32.2)
Brachial approach	5 (4.2)
6Fr guiding	37 (31.4)
7Fr guiding	79 (66.9)
8Fr guiding	2 (1.7)
Use of guide extension catheter	57 (47.5)
Rotational atherectomy*	99 (83.9)
Step up of burr size	10 (8.5)
Used burr size	
1.25 mm	17 (17.2)
1.5 mm	60 (60.6)
1.75 mm	23 (23.2)
2.0 mm	9 (9.1)
Orbital atherectomy	20 (16.9)
Balloon dilatation performed before IVL	29 (24.2)
Max balloon size (mm)	2.22 ± 0.36
Max dilatation pressure (atm)	14.2 ± 3.6
IVL treatment	
Number of catheters per case	1.10 ± 0.30
2.5 mm	44 (36.7)
3.0 mm	53 (44.2)
3.5 mm	23 (19.2)
4.0 mm	12 (10.0)
Total number of pluses	76.5 ± 22.9
Side branch protection	29 (24.2)
Postdilatation before stent deployment	51 (42.5)
Modified balloon	32 (62.7)
Non-compliant balloon	20 (39.2)
Max balloon size (mm)	2.96 ± 0.48
Max dilatation pressure (atm)	16.5 ± 4.8
Drug-eluting stent	
Delivery success	120 (100)
Stent diameter (mm)	3.19 ± 0.51
Total stent length (mm)	36.3 ± 16.0
Post-stent dilatation	95 (79.2)
Max balloon size (mm)	3.45 ± 0.58
Max dilatation pressure (atm)	17.9 ± 4.6

^*^1 case was treated RA followed by OA. *IVL* Intravascular lithotripsy

### Primary safety and efficacy endpoint

The primary efficacy endpoint and the primary safety endpoint were both met in 98.3% cases (95% CI 94.0–99.8 for both). Death and clinically driven revascularization at 30 days were not observed in any patient. Periprocedural MI occurred in 1.7% of cases (Table 3).

**Table 3:** Primary endpoints

Primary endpoints (%: 95% CI)	
Primary safety endpoint Freedom from MACE at 30 days (n = 117)	115 (98.3%: 94.0–99.8)
Cardiac death	0 (0)
Periprocedural MI	2 (1.7)
Spontaneous MI	0 (0)
TVR	0 (0)
Efficacy endpoint Procedural success (n = 118)	116 (98.3%; 94.0–99.8)
Residual stenosis ≥ 50%	0 (0)
In-hospital MACE	2 (1.7)

*MACE* Major adverse cardiac event, *MI* Myocardial infarction, *TVR* Target vessel revascularization

### Angiographic findings

**Fig. 2:**
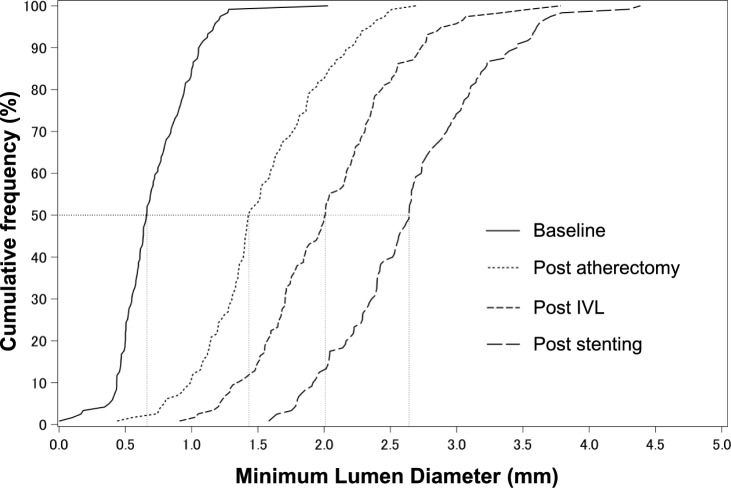
Cumulative frequency curve of minimum lumen diameter by quantitative coronary angiography. Cumulative frequency curves of MLD demonstrating increased lumen gain by each step of atherectomy, IVL, and stent implantation

Angiographic quantitative measurements are shown in supplementary Table 3. The cumulative frequency curves of minimum lumen diameter (MLD) at baseline, after atherectomy, after IVL, and after DES are shown in Fig. 2. MLD increased from 0.72 ± 0.28 mm to 2.66 ± 0.56 mm and %DS decreased from 72.6 ± 9.6% to 15.93 ± 5.6% from pre-treatment to final evaluation, respectively. Final residual stenosis < 30% was achieved in 118 lesions (98.3%). Observed serious angiographic complications at procedure end were limited to 0.8% of cases (Table 4). The main complication was the occurrence of transient slow flow during atherectomy. Deterioration in blood flow after atherectomy resolved in 12 cases, while transient worsening of blood flow after IVL was seen in 2 cases. In these 2 cases, coronary spasm was suspected in the first, while transient blood flow reduction after RA was observed in the second. Another case persisted from after RA to stent placement. No perforation, acute occlusion, or severe dissection occurred during IVL or DES implantation.

**Table 4:** Serious angiographic complications during the procedure

	Post-atherectomy (%)	Post-IVL (%)	Post-stent (%)
Serious angiographic complications	14 (11.9)	3 (2.5)	1 (0.8)
Acute occlusion	0 (0)	0 (0)	0 (0)
Coronary Perforation	0 (0)	0 (0)	0 (0)
Slow flow/no reflow	13 (11.0)	3 (2.5)	1 (0.8)
Severe dissection (Type D to F)	1 (0.8)	0 (0)	0 (0)

*IVL* Intravascular lithotripsy

### OCT/OFDI findings

**Fig. 3:**
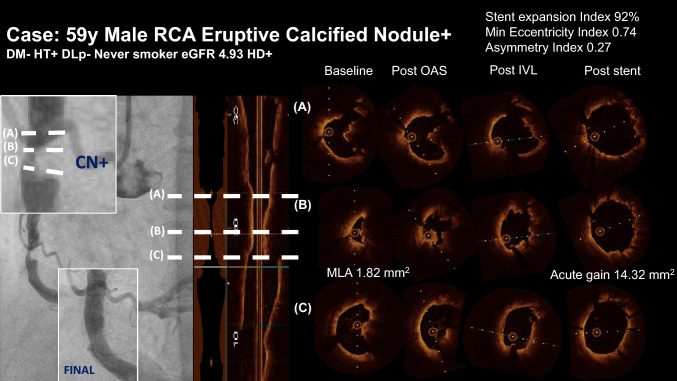
Representative case of combined use of atherectomy and IVL as a lesion preparation of calcified lesion**.** In a 59-year-old hemodialysis patient, an RCA lesion with eruptive calcified nodules was treated with OA and IVL prior to DES implantation; the sufficient DES expansion was achieved with a stent expansion of 92%, an eccentricity index of 0.74, and an asymmetry index of 0.27

Consecutive imaging guidance was performed through pre-atherectomy to post stenting (Supplemental Fig. 1). OCT/OFDI findings are shown in Table 5. At baseline, OCT/OFDI images were obtained from the 68 patients in whom the imaging catheter was able to cross the lesion. Preprocedural MLA was 1.7 ± 0.8 mm^2^. Calcium score at baseline was 4.0 and remained 4.0 after atherectomy in all cases. A total of 109 lesions were assessed by OCT/OFDI after DES deployment. Stent expansion was 81.6 ± 13.5%, and a stent expansion index < 0.8 and < 0.7 was limited to 42.2% and 20.2% of cases, respectively. Eccentricity index was 0.87 ± 0.04, and no patient showed an eccentricity index < 0.7 (Graphical abstract, Fig. 3).

**Table 5:** OCT/OFDI findings

Pre-PCI	*N* = 68
Mean reference lumen area (mm^2^)	7.44 ± 2.86
Minimum lumen area (mm^2^)	1.72 ± 0.76
Calcified plaque at baseline	N = 68
Maximum calcium arc (°)	322.1 ± 53.5
Calcium thickness > 500 μm	68 (100%)
Calcium length (mm)	22.4 ± 11.4
Calcium score	4.0 ± 0.0
Calcified nodule	37 (54.4%)
Post-atherectomy	*N* = 109
Calcium arc (°)	318.3 ± 56.7
Calcium thickness > 500 μm	109 (100%)
Calcium length (mm)	22.4 ± 11.6
Calcium score	4.0 ± 0.0
Calcified nodule	62 (56.4%)
Post DES	*N* = 109
Mean reference lumen area (mm^2^)	8.21 ± 2.80
Minimum stent area (mm^2^)	5.58 ± 2.06
Minimum lumen area acute gain (mm^2^)	4.17 ± 2.01
Stent expansion (%)	81.6 ± 13.5
Stent expansion index < 0.8	46 (42.2%)
Asymmetry index	0.37 ± 0.10
Stent asymmetry > 0.3	78 (71.6%)
Eccentricity index	0.87 ± 0.04
Eccentricity index < 0.7	0 (0%)

*DES* Drug-eluting stent, *OCT/OFDI* Optical coherence tomography/optical frequency domain imaging

## Discussion

This is the first prospective, comprehensive trial of consecutive patients undergoing OCT/OFDI image guidance for PCI to severely calcified lesions treated with a combination of atherectomy and IVL before stenting. The findings confirm the efficacy and safety of combining atherectomy and IVL and emphasize the utility of image guidance in PCI for complex, calcified lesions.

The main findings of this study are as follows:

1. The primary endpoints of efficacy and safety of RA/OA in combination with IVL as a lesion modifier for highly calcified lesions were achieved in 98.3% and 98.3% of cases, respectively;
2. Observed serious angiographic complications mainly followed the atherectomy procedure;
3. OCT findings demonstrated a good final stent expansion index of 0.82 and a limited eccentricity index of 0.87.
4. IVL was selected due to concerns about complications in 42.4% of cases and due to doubts about the effectiveness of additional atherectomy in 60.2%, with some patients having both considerations.

### Study subjects

Inadequate stent expansion is the main cause of DES failure, and lesion calcification is an important determinant of stent failure [2–4]. Recognizing the importance of lesion modification for calcified lesions, various treatments have been investigated for their ability to provide adequate preparation prior to stenting. Until now, however, subjects of these studies have been primarily screened by coronary angiography [20, 23–25]. However, OCT allows substantially more detailed and precise evaluation of calcified lesions than coronary angiography. In particular, a calcification score of 4 by OCT is considered to require specialized lesion modification [26]. Notably, all patients enrolled in our present study met this criterion, even after atherectomy, suggesting the suitability of this cohort for evaluating the efficacy of additional lesion modification by IVL after atherectomy. The frequency of calcified nodules appears to be higher than in the previous reports [27], which may reflect patient background, including a high proportion of hemodialysis patients and high percentage of treatments targeting the right coronary artery; alternatively, it may reflect physician preference for including lesions with no established treatment strategy and historically poor outcomes.

### Combination of atherectomy and IVL

The reported use of combination treatment of atherectomy and IVL in real-world clinical practice suggests that this combined treatment is often necessary for the treatment of complex calcified lesions. Indeed, according to the recently reported REPLICA-EPIC18 trial, which prospectively enrolled 456 patients with IVL, 15.6% were pretreated with atherectomy [28]. The effectiveness of RA is limited in lesions with deep calcification and in cases where guidewire bias does not work effectively. If additional calcified lesion modification is needed for such lesions, IVL—which is not affected by guidewire bias—can be expected to be effective. Additionally, aggressive atherectomy with an increased atherectomy size has not provided a clinical benefit but has been suggested to increase serious complications [10], and the European expert consensus document accordingly recommended a maximum burr/artery ratio of 0.6 during RA procedures [29]. IVL is thought to improve vascular compliance and improve the ability of subsequent balloon inflation to expand the vessel by causing fractures in calcified lesions. It is also known to have a low risk of complications such as slow flow, which was not reported in the DISRUPT CAD III trial [21]. However, one criticism of the currently available IVL platforms is the perceived reduction in ability to cross-complex lesions: long calcified lesions and angulated calcified stenoses may make it difficult for IVL to cross and thus reduce its effectiveness as a therapy. In this study, IVL successfully crossed the target lesion in 100% of patients, even though a 6Fr guide extension catheter was frequently used to cross the lesion after atherectomy. It is worth emphasizing that a potential benefit of the atherectomy-first strategy in this very difficult patient/lesion cohort includes the improved ability to cross the lesion and thus facilitate the easier use of IVL. Thus, the combination of atherectomy and IVL could be considered a strategy that enhances the benefits and mitigates the disadvantages of each when used alone, in either an additive or synergistic fashion.

### Comparison with previous studies

Most of the studies using IVL after atherectomy to date have been limited by retrospective analysis and a small sample size. Nevertheless, their results compare favorably with those for RA alone [30–32]. The recently reported Rota-Shock study was the first prospective observational study in patients with RA and IVL. This study enrolled 160 patients and reported a primary efficacy endpoint of 96.9% [33], which was comparable to the current study. Direct comparison regarding the safety of the procedure is difficult, because the Rota-Shock trial did not evaluate periprocedural MI, the most frequently observed complication in RA cases. Further, the outcome of our study appears to be better than that of the Rota-Shock trial given that coronary perforation was not observed in any cases and that reduced blood flow after IVL was less frequently. Regarding the RA technique employed in the study, RA burr size was similar to that of the Rota-Shock and Prepare-calc studies [24, 34] and can be presumed to be the standard technique. However, the treatment strategy differed from that of the present study. In the Rota-Shock trial, more than half of the patients received IVL after RA failure, including failure due to stent under-expansion, balloon indentation, and crossing failure after RA, etc. The authors reported that coronary dissection occurred more frequently in cases with post-RA balloon or stent crossing failure. In our study, 100% of the patients received IVL electively. Another important difference is the rate of use of intracoronary imaging guidance: in the present study, consecutive image guidance was mandatory (exclusively OCT/OFDI). In the Rota-Shock trial, overall use of imaging (IVUS was used in most of the procedures) represented approximately 50% of cases, but the distribution of imaging was limited to 15–30% from before RA to after IVL. Moreover, the authors of the Rota-Shock study mentioned that a higher proportion of patients who underwent intravascular image-guided procedures were prone to the use of combination of atherectomy and IVL. Thus, image-guided PCI may have facilitated the use of IVL. In this study, 60% chose to add IVL because of the questionable efficacy of additional atherectomy, and 40% because of concerns about complications after RA. Thus, it may be reasonable to consider that differences in PCI strategies, including imaging guidance, may have caused differences in safety concerns.

### Stent expansion

Previous imaging studies have investigated several stent expansion indices to predict stent-related outcomes [35]. Recent guidance for intracoronary imaging has recommended a cut-off stent expansion index value of > 0.8 to indicate optimal stent expansion [5]. In the present study, mean stent expansion index was 0.82 ± 0.14 and stent expansion index < 0.8 was limited to 42.2% of cases. To our knowledge, no treatment strategy has achieved a stent expansion index > 0.8 for calcified coronary lesions. Further, many previous studies which adopted lesion modification prior to stenting for heavily calcified lesions lacked systematic OCT assessment at baseline and after stenting; nevertheless, the reported stent expansion index was approximately 70–75%, and was less than 0.8 in more than 2/3 of cases [21, 22, 36, 37]. The present study confirmed the severity of calcified lesions at baseline and after RA. Therefore, the stent expansion index of > 0.8 we achieved should, therefore, be considered highly meaningful. Such consistent outcomes suggest clinically important efficacy in a wide range of lesion types. Since calcium thickness after atherectomy was greater than 500 μm in every case, this is probably consistent with a previous finding that fracture after IVL occurred regardless of calcification thickness [38]. However, the impact of the stent expansion index on calcified lesions remains unknown, although 1-year follow-up of the patients in this study will elucidate the true clinical impact of a stent expansion index > 0.8 on calcified lesions. Another interesting finding in this study is that an eccentricity index > 0.7 was achieved, suggesting that uniform symmetrical stent expansion was achieved in all cases. Uniform expansion of stents may be also consistent with reports that IVL was effective regardless of calcium thickness. Previous studies have revealed that very high-pressure post-dilatation balloon inflations resulted in more uniform stent expansion compared with RA or modified balloon. However, super high pressure was not applied in any case. Considering that asymmetric stretching of adjacent less-calcified tissues likely contributes to lumen gain in calcified lesions, uniform stent expansion by RA and subsequent IVL without ultra-high-pressure inflation may mean a less traumatic and therefore safer procedure for both the coronary artery and the patient, perhaps hinting at a safer strategy. A comparison of reported stent expansion indices between the present and previous studies is shown in supplemental Fig. 2.

### Clinical implications

The alternative of aggressive RA use has not been shown to be effective [10, 20], although planned RA use is reported to reduce procedure duration and perioperative complications to a greater extent than emergency crossover [37]. Therefore, planned use of RA/OA followed by IVL for severely calcified lesions may be a reasonable approach to increasing procedural success rates and make the procedure safer. Imaging after RA/OA was useful in this study to identify cases in which IVL should be added. Imaging not only confirmed calcification severity and stent expansion, but also identified cases with low efficacy and high risk of aggressive atherectomy treatment. Thus, intracoronary image-guided PCI should be considered, particularly with regard to recent guidance to aid decision-making in the treatment of extensively calcified lesions [12].

### Study limitations

Our results should be interpreted with some important limitations. First, this is a single-arm prospective study. The relatively small sample size and absence of a control group need to be acknowledged, and the need remains to compare the superiority of RA/OA or IVL alone versus RA/OA + IVL. In addition, procedures based on imaging findings were left to the judgment of the attending physician. Therefore, procedures were not uniform, reflecting real-world practice. Second, given the observational nature of our study, a degree of selection and confounding bias cannot be excluded. Third, the size selection of IVL and RA burrs was not specified preoperatively, although the RA burr sizes chosen were, nevertheless, almost identical to those in other studies. Fourth, the preoperative assessment of calcification was not universal as imaging catheters were unable to cross prior to lesion modification. However, a recent study comparing RA and OA with preoperative OCT evaluation of calcified lesions reported that preoperative OCT observation was available in 68% of cases [22], similar to the present study. This is one of the limitations of evaluation of calcified lesions in general and is not specific to our dataset. Fifth, this report was limited to 30 days of observation; and while the 1-year results are awaited; although effectiveness was assessed, cost-effectiveness was not studied.

## Conclusions

Image-guided IVL after atherectomy as a lesion preparation strategy for DES deployment in complex severely calcified lesions demonstrated high procedural success with low MACE rates and allowed optimal stent expansion. Thus, the combination of atherectomy and IVL is a reasonable approach to improving short-term outcomes that can be implemented in clinical practice.

## Supplementary Information

Below is the link to the electronic supplementary material.

Supplementary file1 (DOCX 185 KB)

## Data Availability

Participant data from this clinical trial will not be shared without entering into a contract.

## Abbreviations

DES: Drug-eluting stent
PCI: Percutaneous coronary intervention
OCT: Optical coherence tomography
OFDI: Optical frequency domain imaging
RA: Rotational atherectomy
OA: Orbital atherectomy
IVL: Intravascular lithotripsy
MACE: Major adverse cardiac event
MI: Myocardial infarction
