# Supplementary material for: Dual-Prep registry: atherectomy devices and intravascUlAr lithotripsy for the PREParation of heavily calcified coronary lesions registry
Source: Cardiovasc Interv Ther. 2025 May 12;40(3):553–64. doi: 10.1007/s12928-025-01130-9 (PMC12167257; doi:10.1007/s12928-025-01130-9)
Supplement: Supplementary file 1 — Supplementary file1 (DOCX 185 KB) [file 12928_2025_1130_MOESM1_ESM.docx]

# **Supplemental appendix**

**Dual-Prep Registry：Atherectomy Devices and intravascUlAr Lithotripsy for the PREParation of heavy calcified coronary lesions registry**

Table 1. Study protocol

Table 2. Prespecified endpoint and definition

Table3. Quantitative coronary angiography data

Figure 1. Study flow chart. Consecutive image-guided before, during, and at the end of the procedure of PCI procedure was recommended.

Figure 2. Comparison of reported stent expansion index with previous article of calcified lesion treated with various lesion modification.

## **Table 1**

| **Item** | **Details** |
| --- | --- |
| Study design | A prospective multicenter study of Japanese patients who underwent PCI with atherectomy and IVL prior to DES for severely calcified lesions. |
| Enrolment period | Between November 2023 to June 2024 |
| Setting | 20 Japanese institutions, nationwide. Patients were followed-up as part of routine clinical practice. Patients were expected to visit the hospital at 30 days (can be done by phone) and 12 months (can be done by phone) after discharge. |
| Inclusion criteria | - Age ≥18 years - Consent to participate in this study - Severely calcified lesions where a combination of atherectomy and IVL is desirable as a lesion modification |
| Exclusion criteria | - Patients on follow-up from this study, or who are participating/may participate in another clinical study that may affect the results of this study. - Patient ineligible for the treatment with atherectomy and IVL |
| Primary safety endpoint | Freedom from major adverse cardiac events (MACE) within 30 days of the index procedure. |
| Primary effectiveness endpoint | Procedural Success is defined as stent delivery with a residual stenosis <50% (angiographic core laboratory-assessed) and without in-hospital MACE. |
| Sample size | The required sample size for the registry was calculated based on the incidence of MACE at 30 days. In general, studies using atherectomy devices have reported a higher incidence of intraoperative complications such as perforation and slow flow/no reflow compared to conventional PCI. In addition, the study included patients in real clinical settings requiring atherectomy and IVL. Considering these conditions, the 95% confidence interval is 82.4-94.7% if the MACE-free rate, the primary safety endpoint, is 90% for the enrollment of 110 patients (assuming a 10% dropout rate). A literature review comparing the results of this study with previously reported clinical outcomes using atherectomy devices and IVL systems is warranted. |
| Principal investigator | Masato Nakamura, Toho University Ohashi Medical Center |
| Imaging analysis-responsible investigator | Yohei Sotomi, Graduate School of Medicine, Osaka University |
| Site monitoring: | Micron Inc, Tokyo, Japan |
| Data analysis and biostatistics | Osaka University, Osaka, Japan |
| Clinical Events Committee: | Yuji Ikari, Tokai University, Kanagawa, Japan |
| Angiographic and OCT core laboratory: | Micron Inc, Osaka, Japan |

**Study sites and Principal Investigators:** Miyazaki Medical Association Hospital, Nehiro Kuriyama; Shonan Kamakura General Hospital, Yutaka Tanaka; Sapporo Higashi Tokushukai Hospital, Seiji Yamazaki; Shin Koga Hospital, Tomohiro Kawasaki; Fujita Health University, Takashi Muramatsu; Kurashiki Central Hospital, Kazushige Kadota; Japanese Red Cross Musashino Hospital, Takashi Ashikaga; Sakurakai Takahashi Hospital, Akihiko Takahashi; Higashi Takarazuka Satoh Hospital, Satoru Otsuji; Kokura Memorial Hospital, Kenji Ando; Iwate Medical University Hospital, Masaru Ishida; Kyoto Katsura Hospital, Sigeru Nakamura; Saiseikai Yokohamashi, Yoshiaki Ito; Toho University Ohashi Medical Center, Masato Nakamura; Kindai University Hospital, Gaku Nakazawa; Osaka Saiseikai Nakatsu Hospital, Junya Shite; Kikuna Memorial Hospital, Junko Honye; Kitasato University Hospital , Junya Ako; Fukuoka Sanno Hospital, Hiroyoshi Yokoi; Teikyo University Hospital, Ken Kozuma

## Table 2 Pre-specified endpoints and definitions

| **Items** | **Definition** |
| --- | --- |
| Primary safety endpoint | Freedom from major adverse cardiac events (MACE) within 30 days of the index procedure. |
| Primary effectiveness endpoint | Procedural Success defined as stent delivery with a residual stenosis <50% (angiographic core laboratory-assessed) and without in-hospital MACE. |
| MACE | Composite occurrence of cardiac death, myocardial infarction (MI), or target vessel revascularization. |
| Death | **Cardiac Death:** Death due to cardiovascular causes (myocardial infarction, heart failure or arrhythmia). Unwitnessed or unknown death will need to be clarified as cardiac death even in patients with potentially life-threatening conditions like cancer or infections.  **Vascular mortality:** death due to non-coronary vascular causes like cerebrovascular disease, pulmonary embolism, rupture of aortic aneurysm, aneurysm dissecans of the aorta or any other vascular cause.  **Non-cardiovascular mortality:** Death not due to any of the aforementioned causes of death including death by sepsis, pulmonary causes, accident, suicide or trauma. |
| Myocardial infarction (protocol definition) | The definition of myocardial infarction in this study follows the American Society for Cardiovascular Angiography and Interventions (SCAI) definition for MI within 48 hours after PCI and the Fourth Universal Definition of Myocardial Infarction definition for spontaneous MI after 48 hours.  **Periprocedural MI**  a) Stable patients (previous negative troponin levels):   - Peak CK-MB >10x or >5x the upper limit of the institutional reference value (ULN) within 48 hours after PCI with the appearance of a new Q wave in at least 2 consecutive inductions or a new, sustained left bundle branch block (LBBB) - Troponin (I or T) measured within 48 hours after PCI >70x or >35x ULN with the appearance of new Q waves in at least 2 consecutive inductions or new persistent LBBB   b) Patients with acute coronary syndrome (previous positive troponin levels):   - CK-MB (or troponin) value increases from the value immediately before PCI by the same absolute increment as the level described above. - CK-MB (or troponin) levels increase in patients with no confirmed stable or decreasing biomarker values - CK-MB (or troponin) value is increased to the value described above plus a new ST elevation or ST depression and further signs that are clinically consistent with myocardial infarction, such as new onset or worsening of heart failure, persistent hypotension, etc.   **Spontaneous Myocardial Infarction:** When it occurs > 48 hours after the PCI and is not associated with the procedure and is defined as:  • Typical increase and gradual decrease of troponin levels with, at least, a value above the 99th percentile of diagnostic value for the specific center and with, at least, one of the following events:   - Symptoms of acute myocardial ischemia. - Development of pathologic Q-waves on the electrocardiogram (ECG). - Changes on the ECG indicative of ischemia (ST-segment elevation or depression). - Evidence as seen on the imaging modalities of new loss of viable myocardium or new segmental contractility disorders consistent with ischemic etiology. - Identification of intracoronary thrombus on the angiography or the autopsy. |
| Target vessel revascularization | Revascularization at the target vessel (inclusive of the target lesion) after the completion of the index procedure. |
| Clinically driven TLR | Revascularization will be clinically driven if the follow-up angiography shows stenosis > = 50% and in the presence of, at least, one of the following factors:  Past medical history of recurring angina presumably associated with the target vessel.  Objective signs of resting ischemia (electrocardiographic changes) or in some ischemia challenge test (or equivalent) presumably associated with the target vessel.  Abnormal results on invasive functional tests (FFR or iFR).  Target lesion is defined as the segment treated 5 mm proximal to the stent and up to 5 mm distal to it. |
| Angiographic success (<50% residual stenosis) | Stent delivery with <50% residual stenosis and without serious angiographic complications. |
| Angiographic success (≤30% residual stenosis) | Stent delivery with ≤30% residual stenosis and without serious angiographic complications. |
| Procedural success | Stent delivery with a residual stenosis ≤ 30% (core laboratory-assessed) and without in-hospital MACE. |
| Serious angiographic complications | Severe dissection (Type D to F), perforation, abrupt closure, and persistent slow flow or persistent no reflow. |
| Target lesion failure (TLF) | Cardiac death, target vessel myocardial infarction (Q wave and non-Q wave), or ischemia-driven target lesion revascularization (ID-TLR) by percutaneous or surgical methods at 30 days, 6, 12 and 24 months. |
| Stent thrombosis | Stent thrombosis (ARC definite, probable, definite or probable) |
| BARC bleeding | Major bleeding: BARC3,5 |
| Coronary dissection | Classification of Dissection (by the National Heart, Lung, and Blood Institute - NHLBI) |
| Coronary calcification | Angiography shows radiopaque images without cardiac motion prior to contrast injection involving both sides of the arterial wall in at least one location with ≥ 15 mm total calcification length and extending partially into the target lesion.  OCT/OFDI show a) > 180 degrees maximum calcification angle and b) > 5 mm calcification length or c)> 0.5 mm maximum calcification thickness. |
| Expansion index | Expansion index was defined as the ratio of the minimum stent area to an ideal lumen area, calculated via a linear model derived from the proximal and distal reference areas. |
| Asymmetry index | The asymmetry index was defined as (1 − [minimum stent diameter / maximum stent diameter]) in the target area, and an asymmetry value exceeding 0.3 was considered significant. |
| Eccentricity index | The eccentricity index was calculated as the ratio of the minimum to the maximum stent diameter within the same cross-section that exhibited the minimum value throughout the observation area. |
| ARC, Academic Research Consortium; MACE, major adverse cardiac events; TV-MI, target vessel myocardial infarction. | |

## **Table 3. Quantitative coronary angiography data**

|  | Pre | Post Atherectomy | Post IVL | Post stenting |
| --- | --- | --- | --- | --- |
| n=120 | | | | |
| Reference (mm) | 2.67±0.69 | 2.62±0.61 | 2.68±0.58 | 3.16±0.61 |
| MLD (mm) | 0.72±0.28 | 1.54±0.46 | 2.04±0.54 | 2.66±0.56 |
| %DS | 72.6±9.6 | 40.3±16.2 | 23.6±14.4 | 15.9±5.6 |
| Acute gain (mm) | - | 0.82±0.43 | 1.33±0.56 | 1.95±0.56 |
| Residual stenosis>30% |  |  |  | 2(1.7) |
| MLD: minimum lumen diameter, Acute gain=post-pre MLD at each step | | | | |

## **Figure 1.**


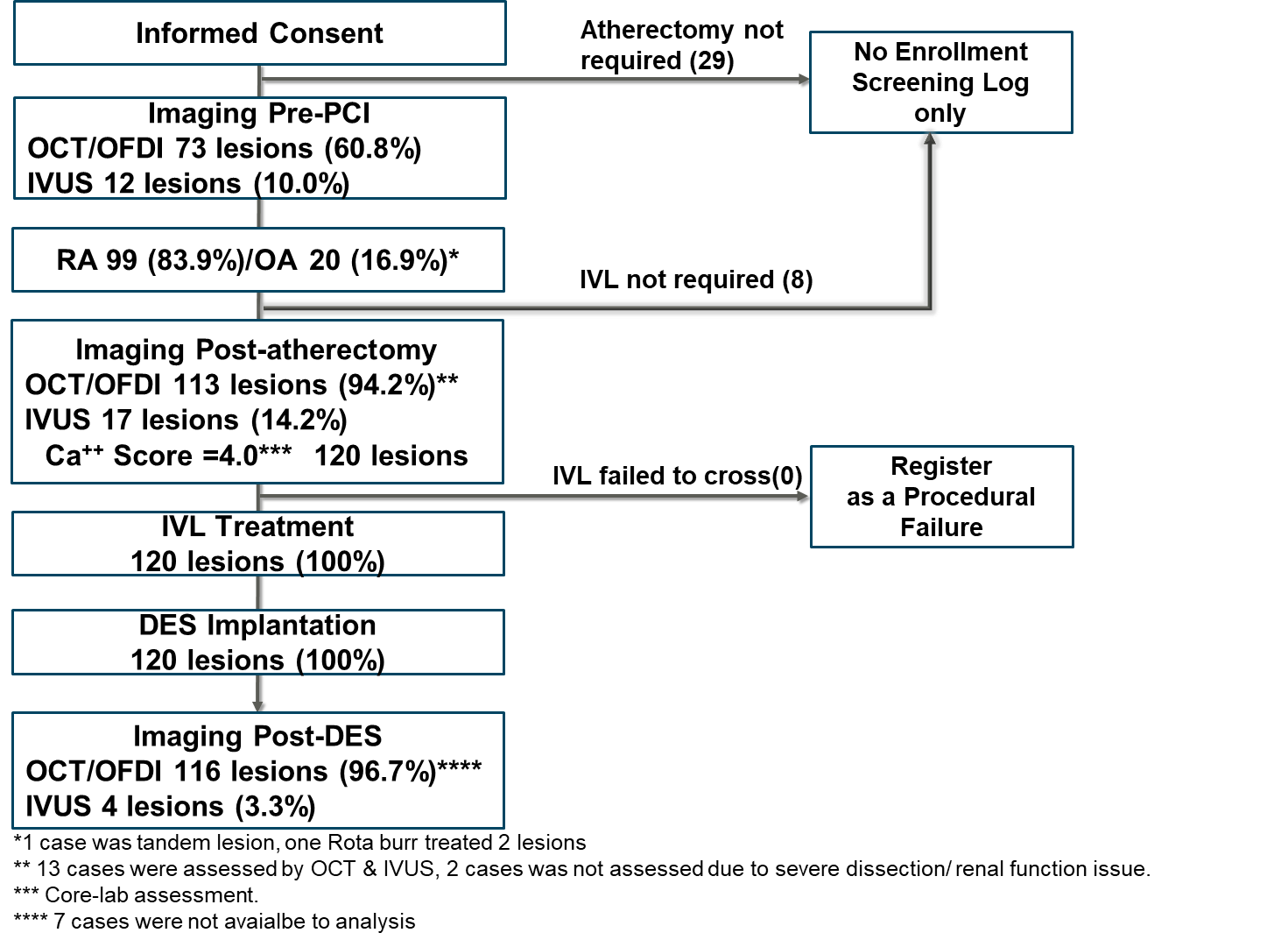


## **Figure 2.**


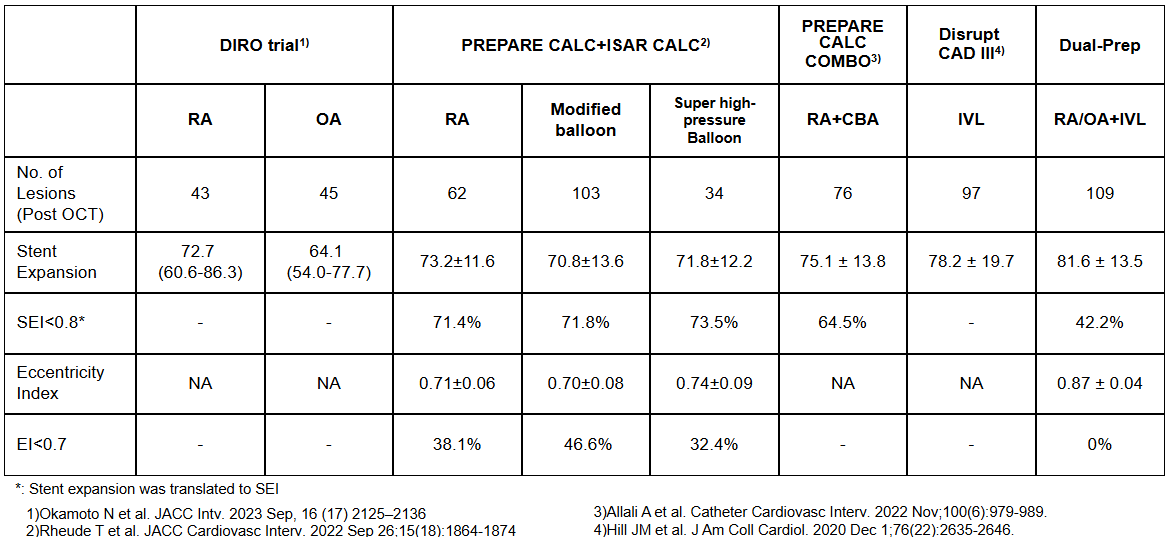


**References**

1. Okamoto N, Egami Y, Nohara H, Kawanami S, Sugae H, Kawamura A, et al. Direct Comparison of Rotational vs Orbital Atherectomy for Calcified Lesions Guided by Optical Coherence Tomography. JACC Cardiovasc Interv. 2023:16:2125-2136.
2. Hemetsberger R, Gori T, Toelg R, Byrne R, Allali A, El-Mawardy M, et al. Optical Coherence Tomography Assessment in Patients Treated With Rotational Atherectomy Versus Modified Balloons: PREPARE-CALC OCT. Circ Cardiovasc Interv 2021:14:e009819
3. Allali A, Toelg R, Abdel-Wahab M, Hemetsberger R, Kastrati A, Mankerious N, et al. Combined rotational atherectomy and cutting balloon angioplasty prior to drug-eluting stent implantation in severely calcified coronary lesions: The PREPARE-CALC-COMBO study. Catheter Cardiovasc Interv. 2022:100:979-989
4. Hill JM, Kereiakes DJ, Shlofmitz RA, Klein AJ, Riley RF, Price MJ, et al. Intravascular Lithotripsy for Treatment of Severely Calcified Coronary Artery Disease. J Am Coll Cardiol. 2020:76:2635-2646.
